# Promising Tensile and Fatigue Properties of Commercially Pure Titanium Processed by Rotary Swaging and Annealing Treatment

**DOI:** 10.3390/ma11112261

**Published:** 2018-11-13

**Authors:** Mingsai Wang, Yanfei Wang, Aihui Huang, Lei Gao, Yusheng Li, Chongxiang Huang

**Affiliations:** 1 School of Aeronautics and Astronautics, Sichuan University, Chengdu 610065, China; mingsaiwang@stu.scu.edu.cn (M.W.); yfwangscu@163.com (Y.W.); aihuihuang@stu.scu.edu.cn (A.H.); lei.gao@scu.edu.cn (L.G.); 2 Nano Structural Materials Center, School of Materials Science and Engineering, Nanjing University of Science and Technology, Nanjing 210094, China; liyusheng@njust.edu.cn

**Keywords:** strength and ductility, fatigue, pure titanium, texture, rotary swaging

## Abstract

The effect of the grain refinement and texture on tensile and fatigue properties in commercially pure titanium (grade 2) processed by rotary swaging (RS) and an annealing treatment is investigated. The as-processed sample consists of band-like grains on the longitudinal section and equiaxed grains on the transversal section and revealed an obvious <10-10> fiber texture with respect to the rod axis. Through this technique, a sample with a high tensile strength of 870 MPa, a high uniform elongation of 8.5%, and a high fatigue limit of 490 MPa can be achieved, and the tensile and fatigue properties are almost the same as those of a conventional Ti-6Al-4V alloy. The enhanced mechanical properties and plastic deformation mechanism are discussed in terms of the observed ultrafine-grained microstructure and strong fiber texture.

## 1. Introduction

For decades, titanium alloys have been of increasing importance in various areas such as the biomedical, aerospace, and automotive industries as well as in other specialized applications, due to their good combination of high biocompatibility, high strength-to-weight ratio, good mechanical properties, and excellent corrosion resistance [1,2,3,4,5,6]. Ti-6Al-4V is a general surgical material, but it is widely acknowledged that both the Al and V elements are capable of introducing long-term health problems [5]. In addition, due to the stress shielding effect introduced by the incompatibility in elastic modulus between the implanted alloy and the adjacent bone tissue, the former usually exhibits an insufficient loading capacity. The pure Ti, which has a relatively low Young’s modulus, contains no harmful elements. More importantly, the pure Ti is intrinsically biocompatible and exhibits excellent bone apposition. Therefore, commercially pure titanium (CP-Ti) has been considered as an alternative material in medical applications, such as dental implants, orthopedic implants, and cardiovascular stents [7,8,9]. However, the low strength, limited ductility, and low fatigue strength of CP-Ti restrict this possibility. Severe plastic deformation (SPD) can significantly increase the tensile strength of metallic materials through grain refinement, according to the Hall‒Petch relationship. Nowadays, there is a body of research into the deformation behavior of ultrafine-grained (UFG) metals processed via equal channel angular pressing (ECAP), high-pressure torsion (HPT), hydrostatic extrusion (HE), rotationally accelerated shot peening (RASP), accumulative roll bonding, etc. [10,11,12,13,14,15,16]. In particular, a complex SPD processing method consisting of ECAP and other plastic deformation (such as rolling and extrusion) can enhance the yield strength further up to 1 GPa for CP-Ti [17,18]. However, the low ductility of these bulk UFG Ti samples prevents them from being used in practical applications.

Several strategies have been proposed to improve the ductility of UFG CP-Ti without sacrificing its strength. For instance, a combination of warm ECAP and cold extrusion followed by annealing treatment can improve the tensile strength up to 1050 MPa, with a total elongation of 8% [18]. Cryorolling followed by annealing treatment produced a fine structure consisting of bimodal/multimodal grain size distributions. This structure reveals a combination of a high yield strength of 926 MPa and a large uniform elongation of 11% [19]. More recently, asymmetrical rolling and subsequent partial recrystallization produced a heterogeneous lamella (HL) structure, which can unite the high strength of ultrafine grains and the high ductility of coarse grains [20]. Compared with the properties of those manufactured by traditional processing technologies, tensile strengths of CP-Ti parts processed by selective laser melting have been improved to 757 MPa while maintaining the total elongation of 19% [21]. By taking into account the strength, ductility, and product dimension, there are still major challenges associated with these techniques for mass production on an industrial scale.

As to the fatigue properties, several studies have revealed an enhanced high cycle fatigue (HCF) limit of UFG CP-Ti. The highest HCF limit of 460 MPa was reported in UFG CP-Ti (grade 2), produced by ECAP [22,23,24]. It was observed that the fatigue crack initiation was significantly delayed in the transverse direction due to the strong crystallographic texture of UFG Ti fabricated by accumulative roll bonding (ARB), while the crack propagation behavior was not obviously affected [25,26,27]. Although these studies revealed some interesting fatigue behaviors of UFG CP-Ti, investigations into the fatigue properties of UFG CP-Ti are still limited.

In this work, we use the technique of rotary swaging (RS) and annealing treatment to achieve a high-performance CP-Ti with an ultimate tensile strength of 870 MPa, uniform elongation of 8.5%, and fatigue limit of 490 MPa. A UFG-like microstructure with preferred orientation was revealed to be responsible for such high performance.

## 2. Experimental Procedures

A commercially pure titanium (grade 2) was used as the studied material. A hot extruded bar in diameter of 32 mm was severely deformed by rotary swaging (RS) at room temperature (RT). The cross-section area reduction from 32 mm to 8 mm resulted in a strain of 2.77, according to *ε* = ln(*A*_0_/*A*), where *A*_0_ and *A* are the initial and final cross section area of samples, respectively [28]. Thereafter, the rods in the as-deformed state were annealed at 450 °C and 500 °C for 5 min, which were labeled as RSA450 and RSA500 samples, respectively.

The microstructures were observed by transmission electron microscope (TEM, Tecnai G2 20, FEI, Hillsboro, OR, USA) operating at 200 kV. The as-received TEM samples with a thickness of about 0.5 mm were cut from the RS-processed rods along the transversal and longitudinal sections, respectively. The slices were mechanically ground to a thickness of 60 μm and finally thinned by twin-jet polishing with a solution of perchloric acid (5%), butanol (35%) and methanol (60%). Electron-backscattered diffraction (EBSD) was carried out on a scanning electron microscope (SEM, Quant 250 FEG, FEI, Hillsboro, OR, USA) in order to examine the crystallographic texture. The step size was 100 nm, and the scanning voltage was 15 kV.

Dog-bone shaped tensile specimens with gauge size of 12 × 2 × 1 mm^3^ were cut from the RS-processed rods along the axial direction. Uniaxial tensile tests were performed at a strain rate of 5 × 10^−4^ s^−1^ at room temperature on a testing machine (BOSE ElectroForce 3230 DMA, Shimadzu, Tokyo, Japan). An extensometer was used to calibrate the strain during tension and each test was repeated for at least three samples. In order to evaluate the fatigue life processed by RS and annealing, high-cycle fatigue (HCF) tests were carried out using smooth funnel-shaped samples, on a high-frequency fatigue machine (QBG-100) at room temperature. The specimens were subjected to a symmetric load (R = −1) under axial loading with the controlled stress. The applied axial load was in a sinusoidal mode with a frequency of 80 Hz.

## 3. Results and Discussion

### 3.1. Tensile Properties

**Figure 1:**
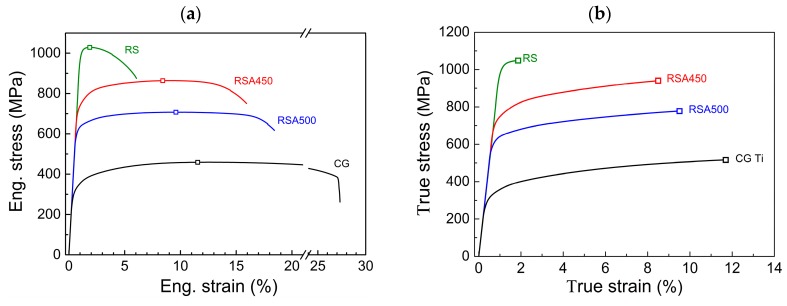

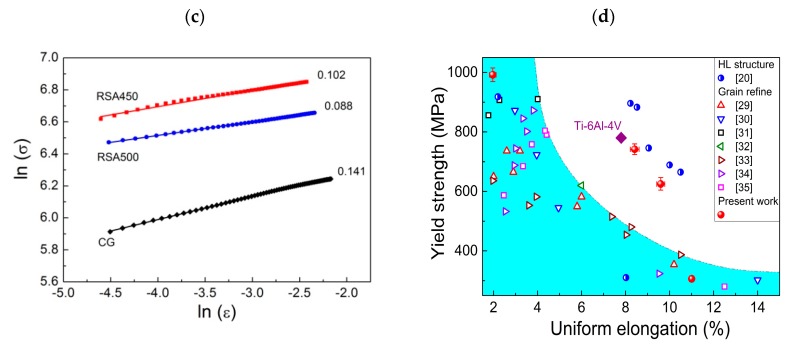
Tensile behaviors of CP-Ti processed by RS and annealing treatment: (**a**) typical tensile curves, (**b**) true stress-strain curves, (**c**) plot of ln(*σ*) − ln(*ε*) for indexing strain hardening exponent in the Hollomon equation (*σ = kε^n^*), (**d**) Excellent strength-ductility synergy of current CP-Ti compared with other experimental results.

Figure 1a displays the typical tensile curves of RS-processed samples compared with their coarse-grained (CG) counterparts. The measured yield strength (YS) and ultimate tensile strength (UTS) of the as-received RS sample were 998 MPa and 1040 MPa, respectively, which are almost three times as high as for the CG sample. This sample loses ductility quickly due to its low strain hardening ability. However, by means of a short (5 min) annealing at 450 °C, a remarkable uniform elongation of 8.5% is regained. Meanwhile, the YS and UTS are still kept at high levels of 740 MPa and 870 MPa, respectively. These excellent tensile properties are almost the same as those of Ti-6Al-4V alloy (see Table 1) and offer a wide potential for large-scale industrial application [22]. Further increasing annealing temperature to 500 °C results in a significant decrease (>100 MPa) in strength, but only a slight increase in uniform elongation. Figure 1b shows the true stress-strain curves converted from those in Figure 1a using a standard formula, demonstrating an obvious strain hardening responsible for ductility. Based on the Hollomon equation ($\sigma =K\varepsilon^{n}$), the strain hardening exponents (*n*) are calculated to be 0.102 and 0.088 for the RSA450 and RSA500 samples, respectively, as shown in Figure 1c. It is only slightly lower than that (0.141) of CG counterpart. Therefore, the RSA450 and RSA500 samples can yield a high uniform ductility very close to its CG sample (11.5%) even at a high stress level.

Figure 1d summaries the yield strength and the uniform elongation of the present samples and those reported in literature for CP-Ti grade 2 [20,29,30,31,32,33,34,35]. In general, the higher the YS, the lower the uniform ductility. For most UFG and NS CP-Ti produced by SPD method, their uniform elongation is usually lower than 5%, while the YS can be much higher than 700 MPa [31,34]. This severely hinders their industrial application. Recently, a novel HL structure by embedding soft micro-grained lamellae into a hard UFG lamellae matrix was fabricated by using asymmetric rolling and subsequent partial recrystallization, which can unite the high strength of UFG and the high ductility of CG [20]. And the data combining high YS and uniform elongation jump out of the regular trade-off region, as shown in Figure 1d. The current RS and annealing process exhibit similar potential to strengthen the CP-Ti with only a limited sacrifice of ductility. It can be seen in Figure 1d and Table 1 that the combination of YS and uniform elongation of current RS-processed CP-Ti reaches the level of tensile properties of Ti-6Al-4V and HL-Ti.

### 3.2. Fatigue Limit

**Figure 2:**
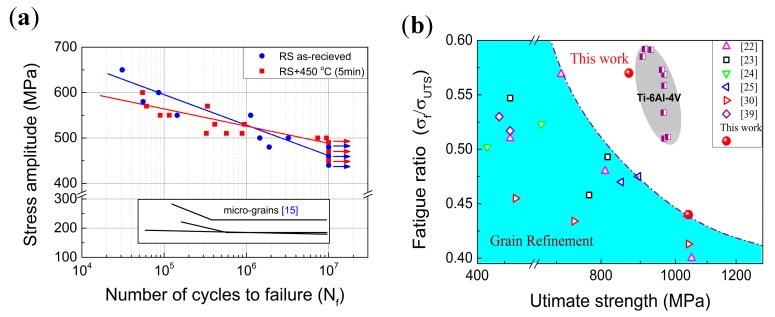
(**a**) Dependence of the fatigue life (*N_f_*) on the cyclic maximum stress for RS and RSA450 samples; (**b**) tensile strength versus fatigue ratio for experimental data of CP-Ti grade 2 and data in the literature and Ti-6Al-4V alloy.

A good fatigue limit of current RS-processed Ti can be expected since the HCF limit of metallic materials is mainly controlled by static strength [36,37,38]. Figure 2a shows the typical S‒N curves of the RS-processed samples. For comparison, the curves of CG CP-Ti are also included [22]. The fatigue limit is defined as the stress at which the sample is deformed for 10^7^ cycles under fully-reversed cycles without failure. It is measured that the fatigue limit of the RS sample in the as-received state is 460 MPa, which is double that of its CG counterpart. Surprisingly, the annealing treatment at 450 °C for 5 min enhances the fatigue limit further to 490 MPa, although the tensile strength is lowered by 170 MPa from the as-received state. This measured fatigue limit is higher than that of most UFG CP-Ti (grade 2) produced by ECAP and ARB methods and approaches that of Ti-6Al-4V alloy [22,23,24,25], as seen in Table 2. The fatigue ratio (*σ_f_*/*σ_UTS_*) against tensile strength is plotted in Figure 2b. It can be seen that the fatigue ratio decreases with the increase of the strength (usually a decrease in grain size) [38], shaping a trade-off type of relationship. However, the RSA450 sample exhibits a large fatigue ratio (0.56) at a high tensile strength of 870 MPa. One can see that the HCF behavior of the RSA450 sample differs significantly from that of conventional CG and UFG CP-Ti [22,23,24,25,26,27,28,29,30,31,32,33,34,35,39], and is close to that of the Ti-6Al-4V alloy.

The HCF behavior of a material is usually described by the Basquin equation as:

(1) $$\sigma_{a}={\sigma^{\prime }}_{f}{\left(2N_{f}\right)}^{b}$$

where *N_f_* is the number of cycles to failure, *σ_a_* is the stress amplitude, *σ′_f_* is the fatigue strength coefficient and might be related to tensile strength, and *b* is the fatigue strength exponent. It is indicated that *b* could reflect the damage mechanism during fatigue and is influenced by strain localization at the crack initiation stage and the stress gradient at the crack propagation stage [38]. A high absolute value of *b* is favorable for the initiation and propagation of cracks. Therefore, the absolute *b* value of the notched CG-Ti sample is almost twice as high as that of the smooth one [24]. Table 2 represents the calculated *b* values of current RS-processed samples and some other reported CP-Ti samples [24]. It is seen that both RS and ECAP deformation raise the absolute value of *b* significantly compared with that of CG-Ti, suggesting an enhanced stress concentration and strain localization for crack initiation and/or crack propagation due to remarkably decreased grain size. The absolute *b* value of the RS-processed Ti can be reduced from 0.053 in the as-deformed state to 0.034 with a short time annealing, indicating that the initiation and/or propagation of a crack is retarded by an ameliorated environment of strain localization and stress concentration. This is probably the reason for the RSA450 sample exhibiting a higher fatigue limit, though its tensile strength is lower than that of the as-received one.

### 3.3. Microstructures with Preferential Crystallographic Texture

**Figure 3:**
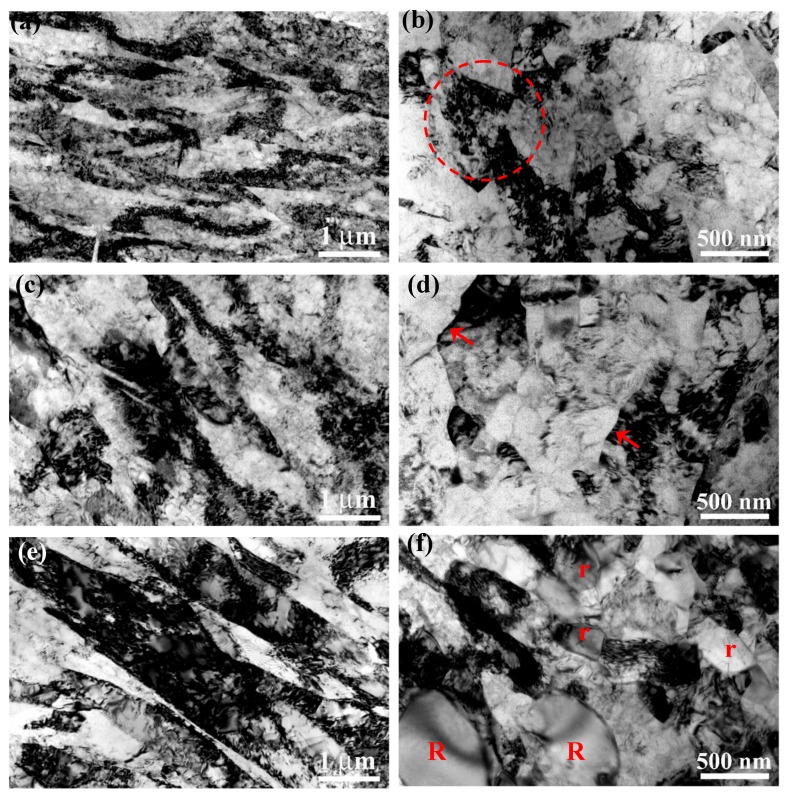
Typical TEM micrographs showing the microstructures on longitudinal section (left side) and transversal section (right side) of the RS-processed samples: (**a**,**b**) as-received, (**c**,**d**) RSA450, (**e**,**f**) RSA500.

The microstructures of the sample after RS deformation and subsequent annealing were investigated by TEM and EBSD in order to reveal the strengthening and toughening mechanisms. Figure 3a–f show the typical TEM micrographs taken from the rods along the longitudinal and transversal directions respectively. The microstructure of the RS sample in the as-received state is a severely deformed structure, which is characterized by elongated bands on the longitudinal section and near equiaxed grains/subgrains on the transversal section, as shown in Figure 3a,b, respectively. It is seen that both band boundaries and grain boundaries are very blurry, and the grain shape is also not clear. The complex contrast within grains indicates high internal stress due to high dislocation density, as marked by the red dotted circle in Figure 3b. The grain/sub-grain size of the transversal section is ranged from 400 nm to 1 μm. By annealing treatment at the 450 °C for 5 min, the hardness of RS450 sample is decreased from 280 ± 6 Hv to 256 ± 8 Hv, and apparent dislocation recovery could be observed in TEM images, as shown in Figure 3c,d. In comparison with the microstructure in as-received state, the dislocation density decreases remarkably and the grain boundaries become sharper, as indicated by the red arrows in Figure 3d. Increasing the annealing temperature to 500 °C, dislocation recovery is more significant and static recrystallization takes place as well, as shown in Figure 3e,f. It is seen in Figure 3e that some band boundaries have evolved into the grain boundaries due to significant dislocation recovery. Partial recrystallization is observed in the transversal section. The recrystallized grains are mostly elliptical in shape with the size of 1–2 μm, as marked with “R” in Figure 3f. In addition, recovery can also result in very fine sub-grains (200–400 nm) with very low dislocation density, as seen the sub-grains marked with “r” in Figure 3f. In sum, apparent dislocation recovery and partial recrystallization occurred during annealing treatment, leading to a significant decrease in dislocation density and formation of very fine-grained structure.

The severe RS deformation introduces a large number of defects (dislocations and grain/sub-grain boundaries) into grains, yielding deformed bands and grains/sub-grains in size of submicron-meter magnitude, which strengthen material significantly according to the Taylor dislocation strengthening mechanism and Hall‒Petch relationship [40]. By annealing treatment, dislocation recovery and partial recrystallization result in a UFG-like microstructure with a low density of dislocations, making Ti strong (Hall‒Petch strengthening) and tough (regaining strain hardening capability), as shown in Figure 1. Moreover, the significant decrease of dislocation density releases internal stress and smooths strain localization, which could delay the initiation and propagation of cracks effectively during fatigue (a small absolute value of *b* parameter in Table 3). Therefore, the fatigue limit of the RSA450 sample is enhanced, rather than lowered, though its strength is decreased compared with that in the as-received state. However, it is still difficult to understand the highly uniform elongation of the RSA450 sample that can be improved only by the dislocation recovery [35]. There would be an additional factor controlling its plastic deformation during uniaxial tension.

**Figure 4:**
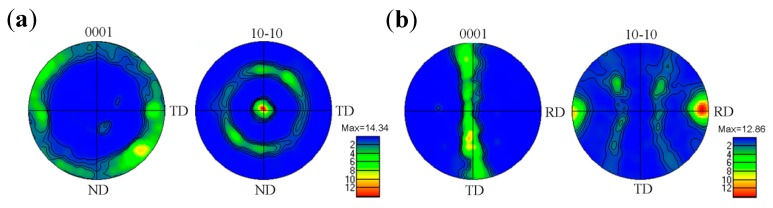
The pole figures for RSA450 sample: basal plane {0001} and prismatic plane {10-10} for transversal section (**a**) and longitudinal section (**b**), the rolling direction alone rod axis, the normal direction and the transverse direction were labeled as RD, ND, and TD, respectively.

Figure 4 shows the representative pole figures for the RSA450 sample obtained from EBSD characterization. For the transversal section, the texture is revealed as a <10-10> fiber texture with respect to the rod axis, while the basal plane is parallel to the rod axis, as shown in Figure 4a. For the longitudinal section, the c-axis deflects from ND to TD uniformly in the range of 0°~90°, while the deflection angle to RD direction is not more than 10°, as seen in Figure 4b. This preferred orientation feature is typical for HCP-Ti produced by swaging and/or drawing [41], suggesting that annealing treatment for a short time does not change the texture formed during RS. It has been revealed that texture plays an important role in the overall mechanical behavior of hexagonal close-packed (HCP) metals [42,43,44,45]. Thus the strong texture would influence the mechanical properties of RSA450 sample and play a different role compared with the ultrafine-grained structure.

**Figure 5:**
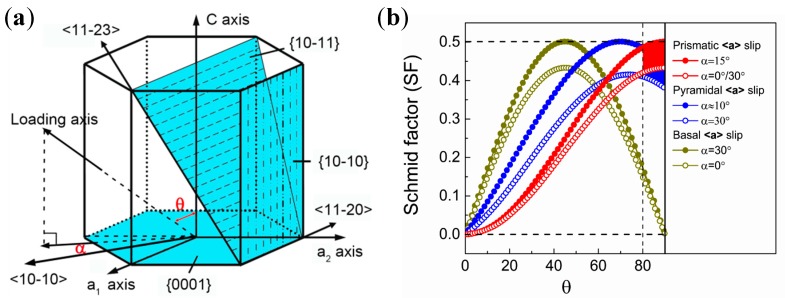
(**a**) Schematic illustration of the dominant texture in the RS-processed sample. Θ: the angle between the c-axis and loading axis; α: the angle between the a-axis and loading axis at the condition where the loading axis is projected onto the basal plane. (**b**) Possible maximum and minimum values of a Schmid factor of three slip systems (prismatic, basal, and pyramidal <a> slips) as a function of the angle Θ.

There are four possible slip systems on three slip planes for pure Ti, including <11-20> slip on the prismatic plane {10-10}, <11-20> slip on the basal plane {0001}, <11-20> slip on the pyramidal plane {10-11} and <11-23> slip on the pyramidal plane {10-11} [41,46], as shown in Figure 5a. Activation of either hard or soft mode of slip is controlled by the loading orientation, i.e., Schmid factor. Figure 5b shows that the curves of the Schmid factor vary with the angle between the c axis and the loading direction in this study. The result is also summarized in Table 3, where the possible maximum and minimum Schmid factors for the three slip systems at two θ angles are given. When the loading direction is parallel to the rod axis, the orientation corresponds to Θ = 90° and it is highly favorable for the prismatic slip {10-10} <11-20> with Schmid factor varying from 0.43 to 0.5, but unfavorable for the basal slip {0001} <11-20> with Schmid factor of 0. At the same time, the Schmid factor of the pyramidal slip {10-11} <11-20> is higher than 0.4, which can also be activated with increasing flow stress. The analysis of Schmid factor indicates that both the prismatic <11-20> slip systems and pyramidal <11-20> slip systems are in soft orientation relative to the tensile axis.

It is clear that the crystallographic texture formed during RS provides an ideal slip mode for the subsequent tensile deformation. If the initial dislocation density on the slip plane is low, abundant dislocation activities (e.g., generation, slipping, interaction, etc.) are anticipated to occur during tension. This is why dislocation recovery after a short annealing can make the RS-processed Ti sufficiently tough, because of the remarkable decrease in dislocation density, as seen in Figure 3. It should be noted that, in contrast to the fine grain size that sharply enhances yield strength, the soft model for dislocation slip would result in a decrease of yield strength due to the relatively low critical resolved shear stress [44,47]. Therefore, there is an equilibrium of strength between the improvement from fine grain size and the drop due to the soft mode of dislocation slip. In the current pure Ti, the UFG-like microstructure enhances strength significantly, while the easy slip mode of dislocation guarantees ductility enough. An enhanced HCF limit of RS-processed CP-Ti is expected because the HCF life is mainly determined by the strength [36,37,38]. As to the effect of texture on fatigue behaviors of UFG Ti, it is revealed that the fatigue crack initiation depends on the loading direction of samples, which obviously could be understood by the ease of the prismatic slip [25]. However, the crack propagation did not apparently depend on sample direction because of mutual cancelation effects of elongated grains and texture [25]. A further study of the influence of texture on fatigue behaviors in UFG Ti is still needed.

## 4. Conclusions

A UFG-like microstructure with strong <10-10> fiber texture was developed in CP-Ti rods by RS deformation and subsequent annealing treatment. When tested along the rod axis, this material exhibits excellent tensile properties with high strength of 870 MPa, high uniform elongation of 8.5%, and a high fatigue limit of 490 MPa. It is found that the refined grain size contributed to the high strength, while the combination of significant dislocation recovery after annealing and the easy slip mode is responsible for the high ductility. Both the tensile and fatigue properties of current RS-processed pure Ti are very close to those of the Ti-6Al-4V alloy, which offers potential for large-scale industrial application.

## Figures and Tables

**Table 1:** Mechanical properties of present RS-processed samples and Ti-6Al-4V alloy.

Materials	Samples	Yield Strength (MPa)	UTS (MPa)	Uniform Elongation (%)	Total Elongation (%)
CP-Ti (grade 2)	CG	318	440	11.5	28
	RS	998	1040	1.6	6
	RSA450	740	870	8.5	17
	RSA500	620	710	9.5	19
Ti-6Al-4V ^[a]^	ASTM F 67	795	860		10

^[a]^ (Ti-6Al-4V alloy for implants or bone).

**Table 2:** Tensile and fatigue properties of RS samples, in comparison with those of the CG samples in CG states from references [22,24].

Materials	Samples	YS (MPa)	UTS (MPa)	*σ_f_* (MPa)	*σ_f_*/*σ_UTS_*	*σ′_f_*	*b*	Reference
CP-Ti (Grade 2)	RS	989	1040	460	0.44	920	−0.053	
	RSA450	740	870	490	0.56	831	−0.034	
	CG	248	418	210	0.50	311	−0.023	[24]
	ECAP	635	669	350	0.52	654	−0.041	[24]
		970	1050	420	0.40			[22]
		800	816	403	0.49			[22]
	ARB	870	895	425	0.47			[24]
		810	850	400	0.47			[24]
Ti-6Al-4V	CG	875	965	515	0.53			[22]

**Table 3:** Schmid factors calculated for RSA450 sample.

Loading Direction	Θ	Prismatic <a> Slip {10-10} <11-20>	Basal <a> Slip {0001} <11-20>	Pyramidal <a> Slip {10-11} <11-20>
//RD	80°	0.42–0.49	0.15–0.17	0.41–0.49
	90°	0.43–0.5	0	0.38–0.44
